# Molecular characterization of early-stage multi-primary lung adenocarcinoma by transcriptome sequencing—a retrospective study

**DOI:** 10.7717/peerj.20617

**Published:** 2026-01-15

**Authors:** Fang Zhang, Guangqiang Zhao

**Affiliations:** 1 Department of Thoracic Surgery, The Third Affiliated Hospital of Kunming Medical University, Yunnan Cancer Hospital, Kunming, Yunnan Province, China; 2 Department of Cardiac Surgery, Yantai Affiliated Hospital of Binzhou Medical University, Yantai, Shandong Province, China

**Keywords:** Multiple primary lung cancer, MPLC, Lung adenocarcinoma, Transcriptomics, *DUOX1*

## Abstract

**Background:**

To investigate the molecular genetic features of multiple primary lung cancer (MPLC) to provide a basis and new methods for its identification, diagnosis, and treatment.

**Methods:**

Transcriptome sequencing (RNA-seq) was performed on 16 tissue samples from eight patients with synchronous multiple primary lung adenocarcinoma (sMP-LUAD) and eight tissue samples from eight patients with single primary lung adenocarcinoma (SP-LUAD). Differentially expressed genes selected by bioinformatic methods were validated in 24 sets of sMP-LUAD and SP-LUAD samples using quantitative reverse transcription polymerase chain reaction (qRT-PCR). Based on The Cancer Genome Atlas (TCGA) database, the differentially expressed genes responsible for the biological behavior of lung adenocarcinoma and their clinical significance were analyzed.

**Results:**

Overall, 194 differentially expressed genes were identified (*P* < 0.05), including 22 up-regulated and 172 down-regulated genes. Two up-regulated (*DUOX1* and *CACNA2D2*) and three down-regulated (*GPX8*, *COL1A2*, and *COL1A1*) genes were selected for validation by qRT-PCR analysis. The qRT-PCR results showed that the expression of *DUOX1* mRNA in the sMP-LUAD group was significantly higher (*P* < 0.05) than that in the SP-LUAD group; mRNA *CACNA2D2*, *GPX8*, *COL1A2*, and *COL1A1* expression in the sMP-LUAD group was not statistically different from that in the SP-LUAD group (*P* > 0.05). Gene ontology (GO) enrichment analysis showed that *DUOX1* mRNA was mainly enriched in the entries of positive regulation of wound healing and oxidation-reduction processes. Kyoto Encyclopedia of Genes and Genomes (KEGG) pathway analysis showed that DUOX1 can promote reactive oxygen species (ROS) production and be related to thyroid hormone production. Furthermore, based on the TCGA database, we analyzed the biological behavior and clinical significance of DUOX1 in lung adenocarcinoma using bioinformatics technology. *DUOX1* mRNA expression was decreased in all stages and pathological subtypes of lung adenocarcinoma (*P* < 0.05). Immune infiltration analysis showed that DUOX1 with mast cells and eosinophils was positively correlated (*P* < 0.05), and Th2 cells were negatively correlated (*P* < 0.05). Logistic regression analysis showed that the expression of *DUOX1* mRNA was significantly correlated with the patient’s age, lymph node metastasis, and pathologic stage (*P* < 0.05). Kaplan–Meier survival plots showed that low DUOX1 expression was not significantly correlated with OS, DSS, and PFI (*P* > 0.05). Univariate and multivariate COX regression analysis revealed that *DUOX1* mRNA could not be used as an independent factor for predicting prognosis (*P* > 0.05). Therefore, we developed a predictive nomogram model combining clinicopathological variables and *DUOX1* mRNA to predict the survival of patients with lung adenocarcinoma.

## Introduction

Over the past century, lung cancer has evolved from an initially rare disease to a leading cause of cancer-related deaths. GLOBOCAN 2020, compiled by the International Agency for Research on Cancer, predicted that there would be approximately 2.2 million new lung cancer cases and 1.8 million deaths worldwide in 2020, making it the second most common malignancy in the world, with the second highest incidence and the highest mortality rate (Sung et al., 2021). Non-small cell lung cancer accounts for approximately 85% of newly diagnosed lung cancers, with adenocarcinomas being the most common histological type; however, its etiology is not fully understood.

With the application of chest examination using high-resolution computed tomography technology and the implementation of early lung cancer identification, the rate of detection of multinodular lesions in the lungs has increased significantly (Chiang et al., 2022). Multiple primary lung cancer (MPLC), intrapulmonary metastasis (IPM), and diffuse pneumonic type lung adenocarcinoma can all manifest as multinodular lesions in the lungs (Detterbeck et al., 2016). MPLC is a specific type of lung cancer characterized by the presence of multiple nodular lesions, including the simultaneous (synchronous, sMPLC) or sequential (metachronous MPLC, mMPLC) appearance of two or more mutually independent primary cancer foci in the lungs of the same patient. The criteria commonly applied used in the clinical diagnosis of MPLC are those established by Martini & Melamed (1975), subsequently modified by the American College of Chest Physicians (ACCP) in 2013 (Kozower et al., 2013), and described in the American Joint Committee on Cancer 8th edition TNM staging manual in 2017 (Feng & Yang, 2019). Most sMPLC tumors share the same histological features, with adenocarcinoma being the most common pathological type (Tie et al., 2021).

It is difficult to distinguish sMPLC from IPM using existing diagnostic criteria; however, several studies have shown that next-generation sequencing (NGS) technology can be used to diagnose and distinguish sMPLC from IPM (Pei et al., 2021; Tian et al., 2023; Li et al., 2022). For example, the analysis of MPLC using whole-genome sequencing or whole-exome sequencing in terms of tumor heterogeneity and genetic evolution has revealed that MPLC has a high incidence of driver mutations, such as EGFR mutations but also a high rate of variation in driver mutations between cancer foci (Wu et al., 2015; Stiles, 2016). Therefore, further research is needed to identify genetic characteristics common to these lesions.

The study of MPLC has mostly started with mutational profiles and little is known about expression profiles (Wu et al., 2020). RNA signaling profiles and proteomics are often used to explain the molecular regulatory mechanisms of disease and may serve as biomarkers for diseases. As the basis and starting point for the study of gene function and structure, the transcriptome plays an important role in our understanding of the development of the body and the onset and progression of disease. Therefore, to explore the common genetic characteristics of MPLC, we analyzed 16 tumor specimens from eight patients with synchronous multiple primary lung adenocarcinoma (sMP-LUAD) and eight samples from eight patients with single primary lung adenocarcinoma (SP-LUAD) by transcriptome sequencing using NGS technology. Differences in gene expression levels between the two groups were determined, with the aim of providing new insights into the molecular features of MPLC, as well as a novel theoretical basis for the diagnosis and treatment of patients with MPLC.

## Materials & Methods

### Patient data

This retrospective study was approved by the Ethics Committee of the Third Affiliated Hospital of Kunming Medical University, China (No. KYLX202163). All specimens were obtained from willing participants who provided written informed consent. The study participants included patients with sMP-LUAD or SP-LUAD who were admitted to the Third Affiliated Hospital of Kunming Medical University, China, and underwent radical lung cancer surgery between January 2021 and November 2022.

The inclusion criteria were as follows: (1) sMP-LUAD or SP-LUAD diagnosed according to ACCP criteria; (2) standardized radical surgery for lung cancer; and (3) postoperative pathological diagnosis of adenocarcinoma. The exclusion criteria were as follows: (1) Incomplete clinicopathological data; (2) patients who received preoperative radiotherapy, chemotherapy, or other anti-tumor treatment; and (3) a history of other tumors or a combination of other tumors.

### Cancer tissue collection

Intraoperatively resected lung cancer tumor tissue samples were collected and within 30 min of tissue release. The cancer tissues were cut into 0.5 cm^3^ pellets and placed in a lyophilization tube with RNAlater Tissue Preservation Solution. The tubes were then transferred to a −80 °C refrigerator for long-term storage.

### Transcriptome sequencing (RNA-seq)

Total RNA was isolated and purified using TRIzol reagent (Invitrogen, Carlsbad, CA, USA), following the manufacturer’s instructions. Using the Ribo-Zero™ rRNA Removal Kit (Illumina, San Diego, CA, USA) ribosomal RNAs were removed while the remaining RNA molecules were fragmented into small pieces under high temperature using divalent cations. Afterwards, the cleaved RNA fragments were reverse-transcribed to create cDNA. After polymerase chain reaction (PCR) enrichment and purification of adapter-ligated fragments, we performed paired-end sequencing on an Illumina NovaSeq™ 6000 (LC Bio) instrument, following the manufacturer’s recommended protocol; the sequencing read length is 2 × 150 bp (PE150).

### RNA-seq data processing and analysis

Initially, Cutadapt was employed to eliminate sequences that exhibited adaptor contamination, low-quality base calls, or ambiguous bases. Following this, the quality of the sequences was evaluated utilizing FastQC (http://www.bioinformatics.babraham.ac.uk/projects/fastqc/). For the alignment of reads to the Homo sapiens reference genome, both Bowtie2 and Hisat2 were utilized. The reads that were successfully aligned from each sample were subsequently assembled with the aid of StringTie (Pertea et al., 2015). Finally, to create a comprehensive transcriptome, transcriptomes from the 24 samples were consolidated using Perl scripts. Following the completion of the final transcriptome, the expression levels of all transcripts were assessed utilizing StringTie and edgeR. StringTie specifically facilitated the evaluation of mRNA expression levels through the computation of Fragments Per Kilobase of exon model per Million mapped reads (FPKM) (Trapnell et al., 2010). Differentially expressed mRNAs were defined as those with log2 (fold-change) >1 or log2 (fold-change) < −1 and (with *P* < 0.05) determined using the R package, edgeR.

### Screening of differentially expressed mRNA

Based on the FPKM values of the mRNA from each sample in the transcriptome sequencing results, the expression levels were categorized into three groups: the low expression (FPKM ≤ 1), moderate expression (1 < FPKM ≤ 3), and high expression (FPKM > 3) groups. The genes whose FPKM values of samples from the sMP-LUAD and SP-LUAD groups belonged to the different expression level groups were selected as differentially expressed genes. According to their *P*-values, the top five differentially expressed genes were selected as target genes.

### Real-time PCR assay

Total RNA was extracted from lung cancer tissues using the MiniBEST Universal RNA Extraction Kit (Takara, Tokyo, Japan), according to the manufacturer’s instructions. RNA concentrations and A260/A280 ratios of samples were measured using a NanoDrop spectrophotometer (Thermo Fisher Scientific, Waltham, MA, USA). RNA was reverse transcribed to synthesize first-strand cDNA using a FastKing RT Kit (with gDNase) (TIANGEN, China). Quantitative real-time polymerase chain reaction (qRT-PCR) analysis was performed using an ABI 7500 Fast Real-Time PCR System (ABI) with Talent qPCR PreMix (SYBR Green) (TIANGEN, Beijing, China). PCR assays were in a total volume of 20 µl, consisting of 40 cycles of the pre-denaturation phase at 95 °C for 3 min, a reaction phase at 95 °C for 5 s, and elongation at 60 °C for 15 s. *GAPDH* was used as an internal control for mRNA analysis. The primer sequences used are listed in Table 1. Relative gene expression levels were analyzed using the 2^−ΔΔCT^ method.

**Table 1:** Primer sequences.

**Gene name**	**Primer sequences**
*DUOX1*	F: 5′-CATCCTGCTCTATGTCCTGCTCATC-3′
	R: 5′-CACGCTGATCTCCACCTTCTTCC-3′
*CACNA2D2*	F: 5′-ACATAGATGAGGTGACACGGAACT-3′
	R: 5′-ACTGAGATTGGCTTGGAGGTAGAA-3′
*GPX8*	F: 5′-CCTGAGGGTCAAGTTGTGAAGTTC-3′
	R: 5′-TTGTCTAACCAGAGCTGCTATGTCA-3′
*COL1A2*	F: 5′-AAGGCATTCGTGGCGATAAG-3′
	R: 5′-AGCGATACCAGGCAGACCTT-3′
*COL1A1*	F: 5′-GTCCCCCTGGAAAGAATGGAG-3′
	R: 5′-GCACCATCCAAACCACTGAAAC-3′
*GAPDH*	F: 5′-GGAGTCCACTGGCGTCTTCA-3′
	R: 5′-TGCTGATGATCTTGAGGCTGTTG-3′

### Xiantao academic tools

We used the cloud dataset from Xiantao Academic (https://www.xiantaozi.com), namely the RNA sequencing data of lung adenocarcinoma from The Cancer Genome Atlas (TCGA) database. After removing duplicate samples and samples without clinical information, a total of 59 normal lung tissues and 516 primary tumor tissues were included for bioinformatics analysis. We used Xiantao academic to visualize differential analysis, relevance to immune infiltration, clinical pathological characteristics, and prognostic value.

### Statistical analysis

RNA-seq data were evaluated using Fastp software (https://github.com/OpenGene/fastp). The statistical power of this experimental design, as calculated using Fastp, was 0.84. Differentially expressed mRNAs with statistical significance (*P* < 0.05) were identified using the R package edgeR (version 4.5.1; R Core Team, 2025). QRT-PCR data are expressed as log2 fold-change values. To compare differences between groups, non-parametric Mann–Whitney U or unpaired *t*-tests were performed using GraphPad Prism 8 software (GraphPad Software, La Jolla, CA, USA). All experiments were performed in three biological replicates. *P*-values < 0.05 were considered statistically significant.

### Data availability

Clinical information data can be found in Table S1. RNA-seq raw data can be found in File S2. The original data of the qRT-PCR verification experiment has been organized into File S1.

**Table 2:** Clinicopathological characteristics of patients with sMP-LUAD and SP-LUAD.

**Patient characteristic**	**sMP-LUAD (*N* = 8)**	**SP-LUAD (*N* = 8)**
Age, years (mean (range))	58.8 [48–70]	60.1 [52–71]
**Sex**		
Male	3	2
Female	5	6
**Smoking history**		
Yes	3	2
No	5	6
**Tumor characteristics**	**sMP-LUAD (*n* = 16)**	**SP-LUAD (*n* = 8)**
**Largest tumor size, × (cm)**		
x ≤ 1	2	0
1 < x ≤ 2	10	4
2 < x ≤ 3	4	4
**TNM stage**		
IA1	2	0
IA2	9	4
IA3	4	4
IB	1	0
**Tumor site**		
RUL+LUL	3	–
RUL+LLL	1	–
RML+LLL	1	–
RLL+LUL	1	–
RLL+LLL	1	–
LUL+LLL	1	–
**Histological type**		
ADC	16	8

**Notes.**

RUL Right upper lobe RML Right middle lobe RLL Right lower lobe LUL Left upper lobe LLL Left lower lobe ADC adenocarcinomas

## Results

### Clinical data

**Figure 1:**
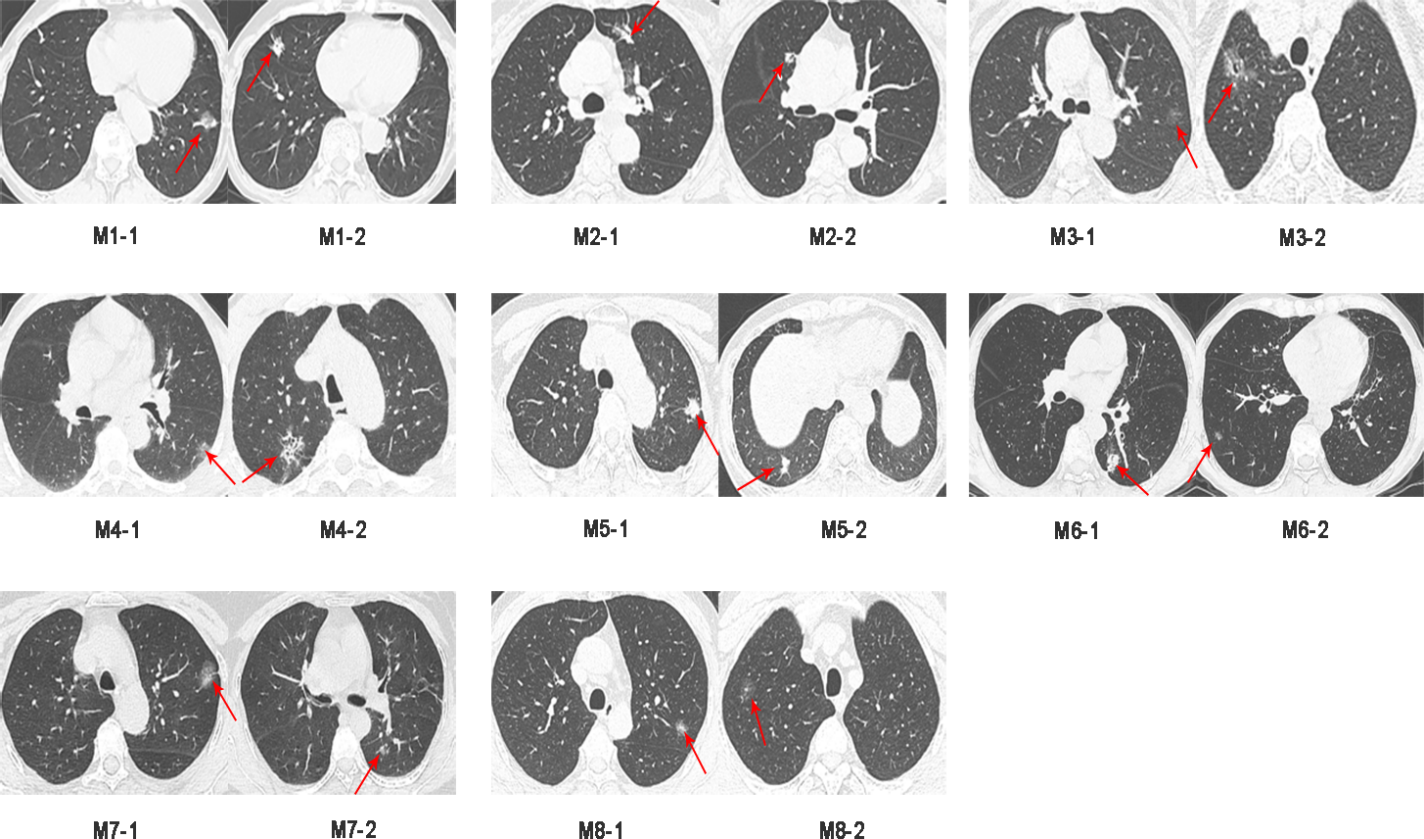
Preoperative CT imaging of the chest in eight patients with simultaneous multiple primary lung adenocarcinoma.

**Figure 2:**
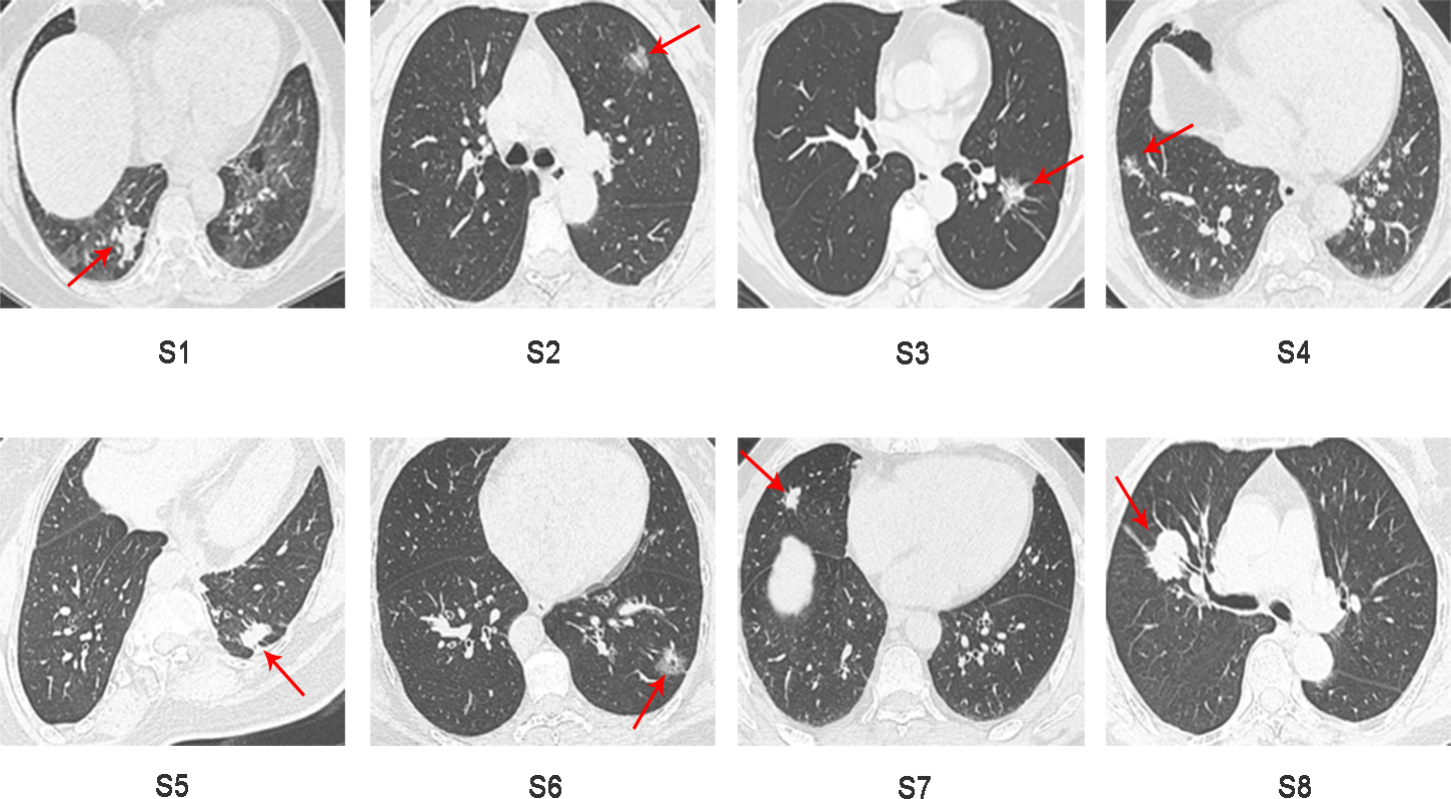
Preoperative CT imaging of the chest in eight patients with independent primary lung adenocarcinoma.

Sixteen surgically resected tumor tissue samples (two main lesions) from eight patients with sMP-LUAD and eight surgically resected tumor tissue samples from eight patients with SP-LUAD were collected according to the enrollment criteria of this study. The participants’ clinical data were analyzed (Table 2), and their computerized tomography (CT) images data are shown in Figs. 1 and 2. All patients were evaluated as having early-stage lung cancer (stage IA1–IB), according to the 8th edition of the TNM staging manual. All tumors were histologically classified as invasive adenocarcinomas. The median age of the patients with sMP-LUAD (five female and three male patients) was 58.8 years, while that of patients with SP-LUAD (six female and two male patients) was 60.1 years. All male patients in this study had a history of smoking, while all female patients had never smoked. In the sMP-LUAD group, the ratio of two samples were collected from each patient located on either sides of the lung was seven out of eight.

### Differential mRNA expression analysis of tumor samples from patients with sMP-LUAD and SP-LUAD

**Figure 3:**
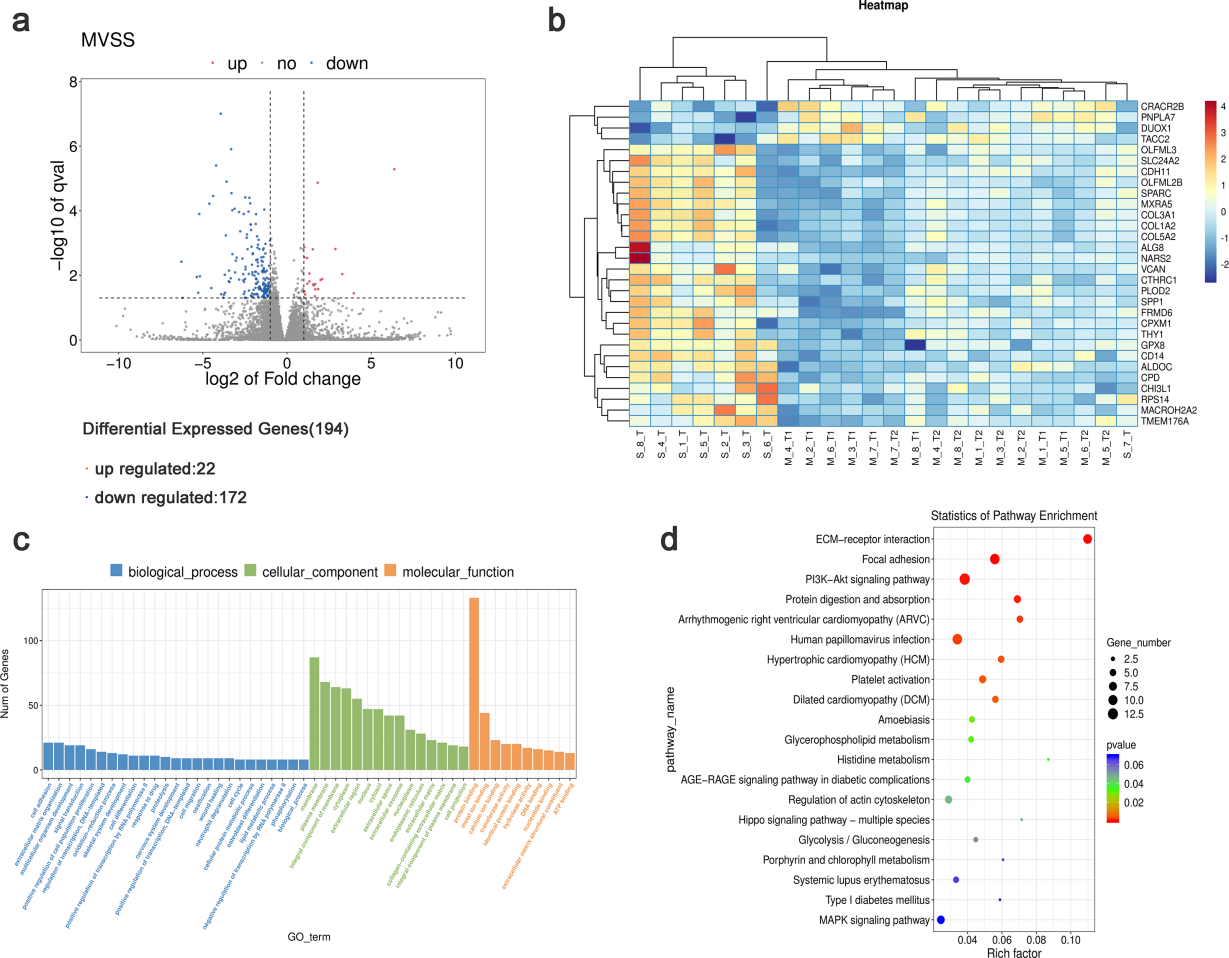
Analysis of differential gene expression levels and enrichment analysis. (A) Volcano plot of deferentially expressed genes in tumors from patients with sMP-LUAD and SP-LUAD. Two vertical lines indicate down-regulation and up-regulation of two-fold difference in expression (log2 transformed), the horizontal line corresponds to a *P* value of 0.05 (−log10 transformed), and the differentially expressed genes are represented by the blue and red dots in the figure. (B) Heat map of deferentially expressed genes in tumors from patients with sMP-LUAD and SP-LUAD showing gene clustering. The horizontal axis represents samples, and the vertical axis represents genes. Red indicates high expression genes, while dark blue indicates low expression genes. (C) GO enrichment analysis of deferentially expressed genes, reflecting the distribution of the numbers of deferential genes enriched in GO terms for biological processes (blue), cellular components (green), and molecular functions (orange). (D) KEGG enrichment analysis. Horizontal axis: enrichment score, the larger the rich factor, the higher the KEGG enrichment level; vertical axis: KEGG metabolic pathway name. The size of the dots represents the number of significantly different genes matched to a single KEGG, and the color of the dots represents the *P* value of the enrichment analysis.

Using StringTie software to compare the gene expression levels in tumor samples from patients with sMP-LUAD and SP-LUAD, a total of 194 differentially expressed genes were identified (*P* < 0.05), including 22 up- and 172 down-regulated genes (Fig. 3A). Clustering analysis revealed that these differentially expressed genes could adequately distinguish between sMP-LUAD and SP-LUAD samples (Fig. 3B). Further, gene ontology (GO) enrichment analysis showed that the differentially expressed genes were mainly enriched in the biological process of cell adhesion; associated with cellular components of the membrane; and were involved in molecular function of protein binding (Fig. 3C). Kyoto Encyclopedia of Genes and Genomes (KEGG) enrichment analysis showed that the differentially expressed genes were enriched in the ECM-receptor interaction and PI3K-Akt signaling pathways (Fig. 3D).

### Detection of differentially expressed genes using qRT-PCR

**Figure 4:**
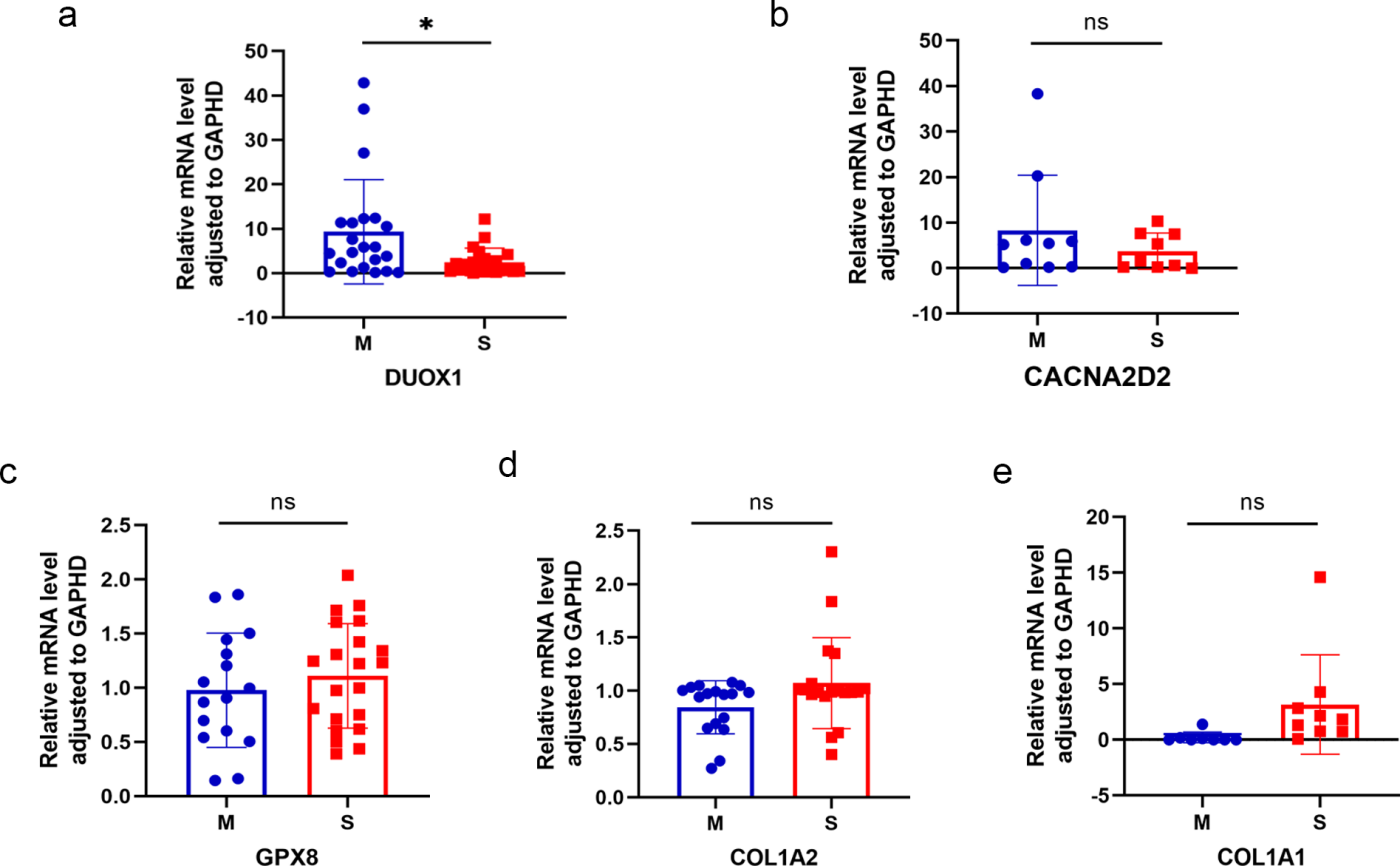
Results of qRT-PCR. Differences in *DUOX1* (A), *CACNA2D2* (B), *GPX8* (C), *COL1A2* (D), and *COL1A1* (E) mRNA levels between SP-LUAD and sMP-LUAD tissues analyzed by qRT-PCR. Blue: synchronous multiple primary lung adenocarcinoma group; red: single primary lung adenocarcinoma group. ns, *P* > 0.05; * *P* < 0.05.

Five differentially expressed genes were selected for subsequent validation using qRT-PCR (Table 3), of which two were up-regulated (*DUOX1* and *CACNA2D2*) and three were down-regulated (*GPX8*, *COL1A2*, and *COL1A1*). Twenty-four pairs of sMP-LUAD and SP-LUAD tissues were collected for qRT-PCR. The results confirmed that the expression of *DUOX1* mRNA in the sMP-LUAD group was significantly higher than that in the SP-LUAD group (*P* < 0.05). The expression of mRNA*CACNA2D2* in the sMP-LUAD group was slightly higher than that in SP-LUAD group; however, the difference was not statistically significant (*P* >  0.05). Additionally, the expressions of mRNA*GPX8*, mRNA*COL1A2*, and mRNA*COL1A1* in the sMP-LUAD group were slightly lower than those in the SP-LUAD group; however, the difference was not statistically significant (*P* > 0.05) (Figs. 4A–4E).

**Table 3:** The FPKM values of DUOX1, CACNA2D2, GPX8, COL1A1, and COL1A2.

**Samples**	**FPKM values**				
	**DUOX1**	**GPX8**	**COL1A2**	**COL1A1**	**CACNA2D2**
M1-1	6.23	0.78	32.38	86.13	1.93
M1-2	5.06	0.90	33.43	42.31	4.10
M2-1	11.38	0.24	17.80	25.55	3.53
M2-2	4.26	0.13	26.76	122.92	4.59
M3-1	25.66	0.34	25.63	30.24	5.27
M3-2	8.88	0.95	27.49	39.34	4.18
M4-1	7.50	0.23	9.91	15.05	0.25
M4-2	2.17	0.90	48.66	93.72	0.03
M5-1	5.60	0.64	16.46	25.55	4.56
M5-2	7.71	1.11	39.75	85.07	5.57
M6-1	7.33	0.35	21.04	32.97	1.72
M6-2	9.23	0.77	30.31	39.55	3.07
M7-1	11.42	0.21	15.32	14.12	3.19
M7-2	6.54	0.30	22.52	60.99	1.37
M8-1	2.85	0.03	29.69	36.00	4.51
M8-2	13.32	0.99	29.02	42.86	3.02
S1	4.33	1.72	86.16	168.58	2.37
S2	2.81	1.61	79.55	80.34	0.32
S3	2.22	5.22	71.28	178.69	0.54
S4	1.39	2.06	82.12	135.80	0.42
S5	3.11	2.01	167.61	322.04	0.74
S6	2.84	3.49	7.11	11.09	0.51
S7	1.71	1.08	40.70	45.06	2.15
S8	0.70	2.97	237.04	479.26	0.88

**Notes.**

FPKM Fragments Per Kilobase of exon model per Million mapped reads

### Comprehensive analysis of DUOX1

**Figure 5:**
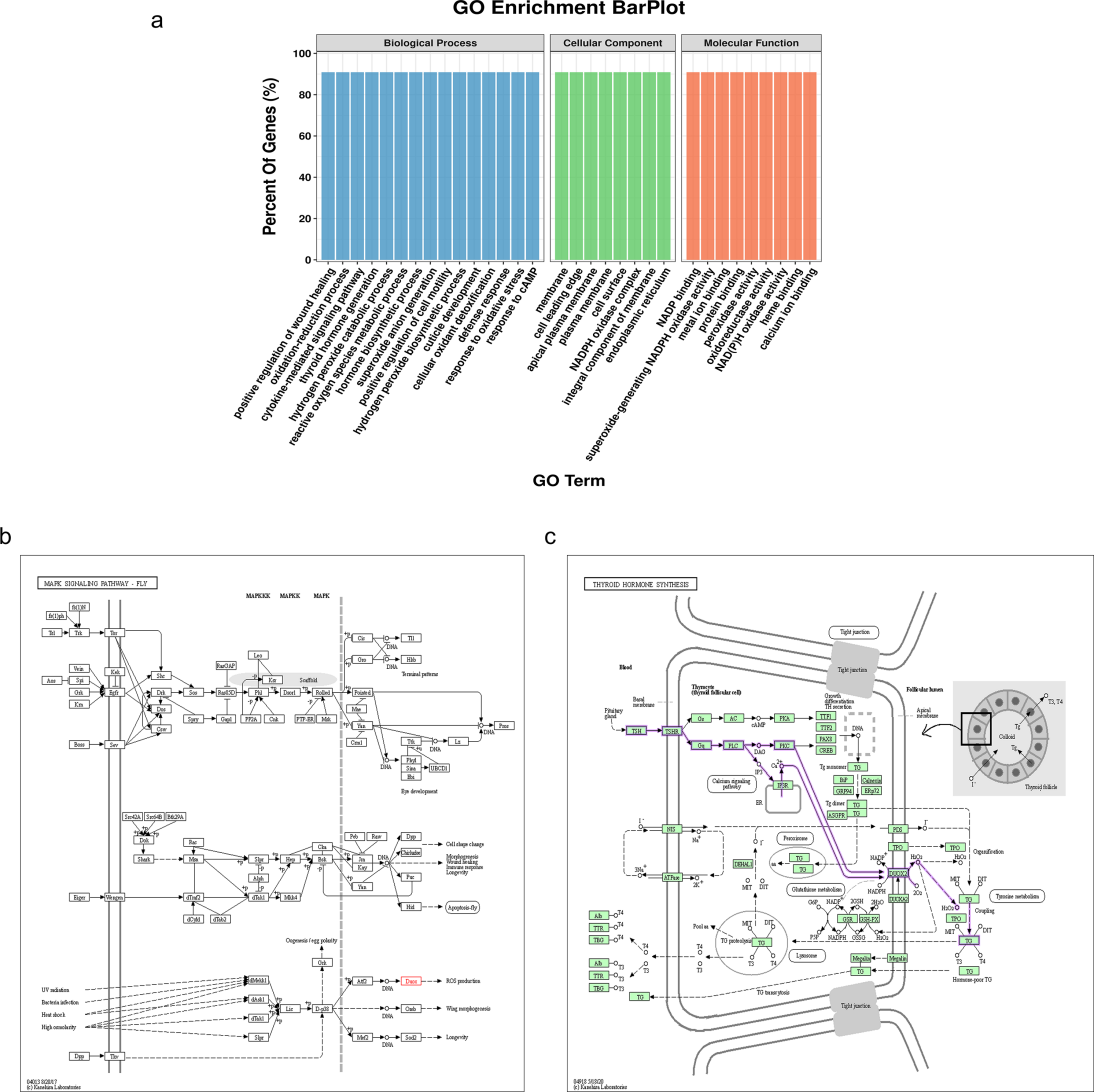
Bioinformatics analysis of the differentially expressed gene, *DUOX1*. (A) GO enrichment analysis of DUOX1 gene. (B) The MAPK signaling pathway involving DUOX1. KEGG PATHWAY: MAPK signaling pathway-fly-reference pathway (C) The thyroid hormone synthesis signaling pathway involving DUOX. KEGG PATHWAY: thyroid hormone synthesis-reference pathway.

The NADPH oxidase, double oxidase 1 (DUOX1), is expressed in well-differentiated primary human respiratory epithelial cells and alveolar epithelial cells. DUOX1 is also a gene differentially expressed between the sMP-LUAD and SP-LUAD groups; therefore, it was annotated using functional analysis, indicating its enrichment in several biological processes, including positive regulation of wound healing and oxidation–reduction processes. Two GO terms indicated that DUOX1 was involved in membrane and protein binding, and its molecular functions overlapped with the dominant classification in the overall GO enrichment analysis (Fig. 5A). KEGG pathway analysis showed that DUOX1 can promote reactive oxygen species (ROS) production (Fig. 5B) and was involved in thyroid hormone production (Fig. 5C).

### Expression pattern of *DUOX1* mRNA in lung adenocarcinoma

**Figure 6:**
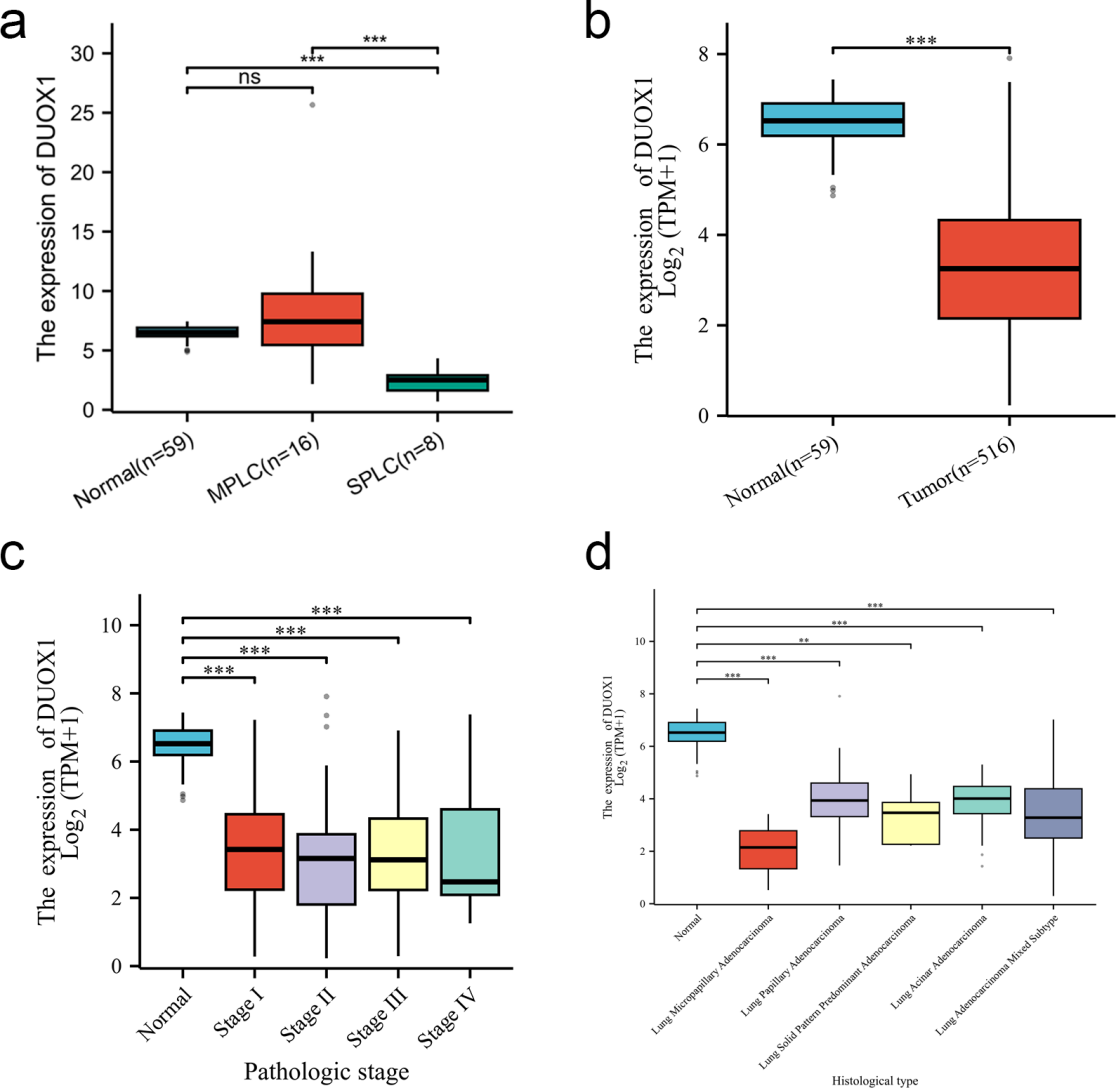
*DUOX1* mRNA is down regulated in single primary lung adenocarcinoma patients. (A) DUOX1 expression between normal, multiple primary lung cancer (MPLC) and single primary lung cancer (SPLC). Based on The Cancer Genome Atlas (TCGA) database, (B) DUOX1 expression between normal and primary tumor tissues; (C) DUOX1 expression in different cancer stages; (D) DUOX1 expression in different histological subtypes. ns, *P* > 0.05, *P < 0.05, **P < 0.01, ***P < 0.001.

We compared the *DUOX1* mRNA levels of MPLC and SPLC in this study with normal tissues in TCGA database. The results showed no significant difference between MPLC and normal tissues (*P* > 0.05), while the *DUOX1* mRNA levels in SPLC were significantly lower than those in normal tissues and MPLC (*P* < 0.05) (Fig. 6A). Based on the TCGA database, the transcriptional levels of DUOX1 in 59 normal lung tissues and 516 primary tumor tissues of patients with lung adenocarcinomas were detected. The results indicated that there was an expression difference between normal tissues and tumor tissues, with the level of *DUOX1* mRNA being decreased in tumor samples (*P* < 0.05) (Fig. 6B). Furthermore, it was observed that DUOX1 expression level was decreased in different tumor stages compared with normal tissues (*P* <  0.05), while there was no difference in expression between the stages (*P* > 0.05) (Fig. 6C). The expression level of DUOX1 was decreased in different histological types of lung adenocarcinoma (*P* <  0.05) and was lower in micropapillary and solid lung adenocarcinoma than in other subtypes (Fig. 6D).

### *DUOX1* mRNA relevance to immune infiltration

**Figure 7:**
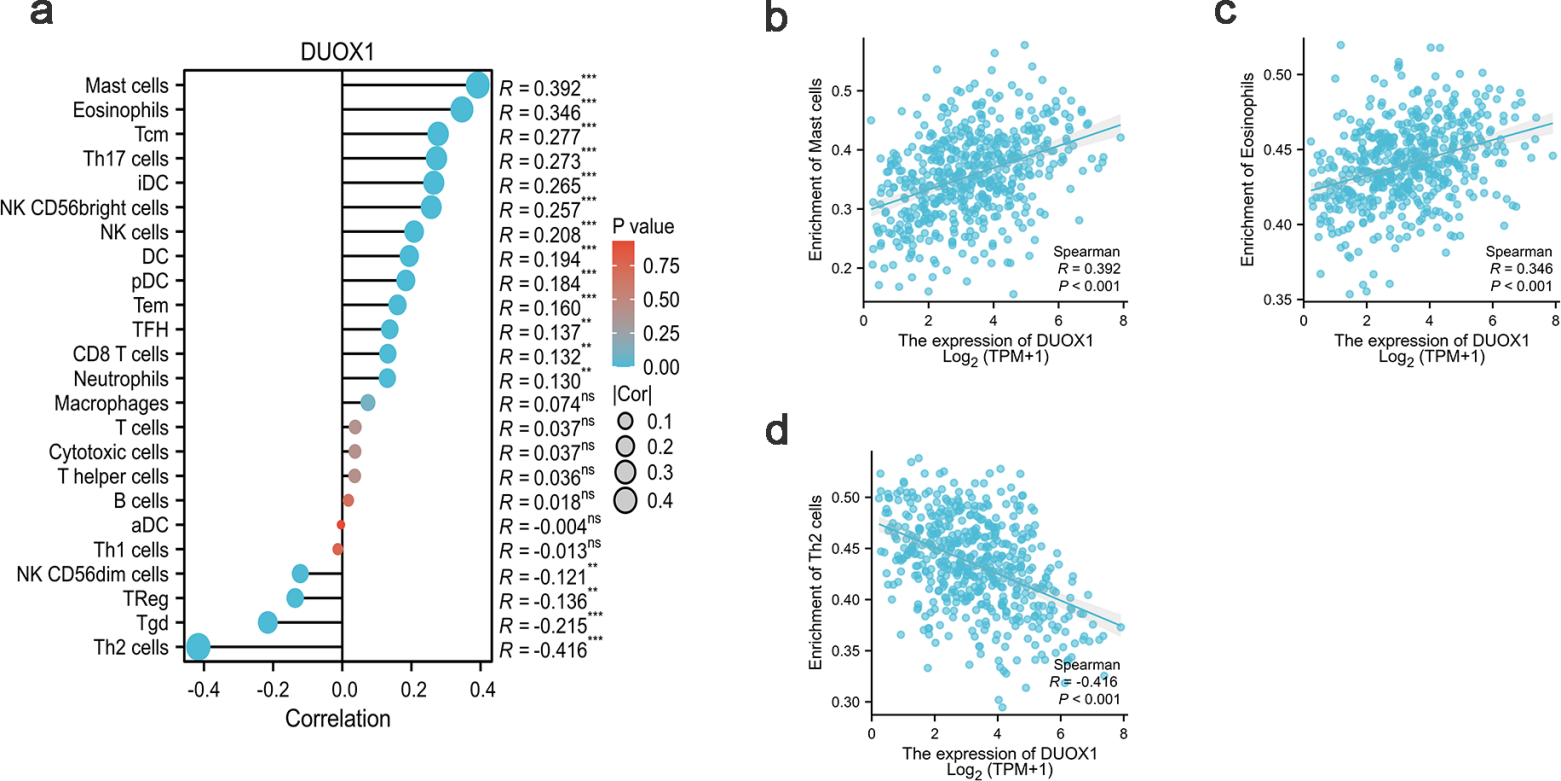
Relationship between DUOX1 expression in the tumor microenvironment of lung adenocarcinoma and immune cell infiltration. (A) Relationship between immune cell levels and *DUOX1* mRNA expression. The relationships between the abundances of (B) mast cells, (C) eosinophils, (D) Th2 cells are shown in scatter plots.

In this study, Spearman correlation was used to show the relationship between *DUOX1* mRNA and the level of immune cell infiltration in lung adenocarcinoma (Fig. 7A). The results showed that DUOX1 expression with mast cells (*R* = 0.392, *P* <  0.001) and eosinophils (*R* = 0.346, *P* < 0.001) was positively correlated (Figs. 7B–7C), while its expression was negatively correlated with Th2 cells (R=−0.416, *P* < 0.001) (Fig. 7D).

### *DUOX1* mRNA expression association with clinical pathological characteristics

In this study, the clinicopathological characteristics of patients with lung adenocarcinoma obtained from the TCGA database were analyzed using logistic regression analysis. The results showed that the expression of *DUOX1* mRNA was significantly correlated with the patient’s age, lymph node metastasis, and pathologic stage (*P* < 0.05). However, there was no significant correlation with gender, smoking status, anatomic neoplasm subdivision, tumor location, tumor size, distant metastasis, and primary therapy outcome (*P* >  0.05) (Table 4).

**Table 4:** mRNA *DUOX1* expression association with clinical pathological characteristics (logistic regression).

**Characteristics**	**Total (N)**	**OR (95% CI)**	*P* value
Age (>65 *vs.*≤65)	497	1.562 (1.096–2.226)	0.014
Gender (Male *vs.* Female)	516	1.133 (0.801–1.602)	0.480
Smoker (No *vs.* Yes)	502	0.855 (0.523–1.398)	0.532
Anatomic neoplasm subdivision (Right *vs.* Left)	501	0.827 (0.578–1.183)	0.299
Location (Peripheral Lung *vs.* Central Lung)	190	1.153 (0.630–2.111)	0.644
Pathologic T stage (T3 & T4 *vs.* T1 & T2)	513	1.316 (0.782–2.215)	0.300
Pathologic N stage (N1 & N2 & N3 *vs.* N0)	504	0.645 (0.445–0.935)	0.021
Pathologic M stage (M1 *vs.* M0)	372	0.837 (0.370–1.896)	0.670
Pathologic stage (Stage II & Stage III & Stage IV *vs.* Stage I)	508	0.682 (0.481–0.969)	0.033
Primary therapy outcome (PD & SD & PR *vs.* CR)	428	0.895 (0.581–1.379)	0.615

### Prognostic value of DUOX1 in lung adenocarcinoma

**Figure 8:**
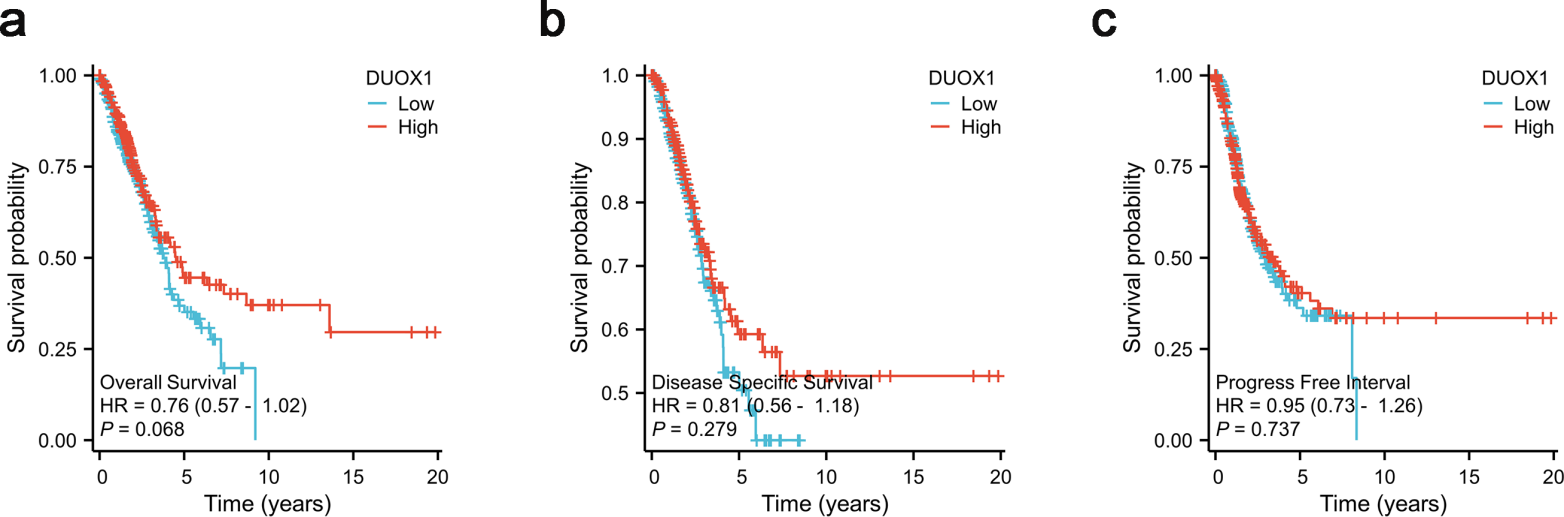
DUOX1 -high and DUOX1 -low groups in lung adenocarcinoma were compared using Kaplan–Meier survival plots with the TCGA database. The analysis included three measures: (A) overall survival, OS, (B) disease-specific survival, DSS, (C) progression-free interval, PFI.

According to Kaplan–Meier survival plots, low DUOX1 expression was not significantly correlated with overall survival (OS) (Fig. 8A), disease-specific survival (DSS) (Fig. 8B) and progression-free interval (PFI) (Fig. 8C) (*P* > 0.05). According to univariate COX regression analysis, DUOX1 low expression had no significant effect on the overall survival of patients with lung adenocarcinoma (*P* > 0.05). T stage (HR = 2.393 [1.641–3.490], *P* < 0.001), N stage (HR = 2.582 [1.922–3.470], *P* < 0.001), M stage (HR = 2.143 [1.251–3.672], *P* = 0.006), pathologic stage (HR = 2.958 [2.177–4.021], *P* < 0.001) and primary therapy outcome (HR = 0.350 [0.249–0.493], *P* < 0.001) increased the risk of death. Multivariate COX regression analysis further showed that DUOX1 low expression did not significantly increase the survival risk of patients with lung adenocarcinoma (*P* >  0.05). T stage (HR = 2.161 [1.170–3.990], *P* = 0.014), N stage (HR = 2.238 [1.071–4.677], *P* = 0.032) and primary therapy outcome (HR = 0.399 [0.281–0.568], *P* < 0.001) were independent risk factors for OS in patients with lung adenocarcinoma (Table 5).

**Table 5:** Univariate and multivariate analyses (overall survival) for prognostic factors in lung adenocarcinoma.

**Characteristics**	**Total (N)**	**Univariate analysis**		**Multivariate analysis**	
		**HR (95% CI)**	*P* value	**HR (95% CI)**	*P* value
**Age**	497				
≤65	239	Reference			
>65	258	1.213 (0.904–1.629)	0.198		
**Gender**	507				
Female	272	Reference			
Male	235	1.070 (0.800–1.431)	0.648		
**Smoker**	493				
Yes	421	Reference			
No	72	1.094 (0.725–1.652)	0.668		
**Anatomic neoplasm subdivision**	493				
Left	196	Reference			
Right	297	1.032 (0.764–1.394)	0.838		
**Location**	183				
Central Lung	63	Reference			
Peripheral Lung	120	0.949 (0.593–1.520)	0.829		
**Pathologic T stage**	504				
T1 & T2	440	Reference		Reference	
T3 & T4	64	2.393 (1.641–3.490)	<0.001	2.161 (1.170–3.990)	0.014
**Pathologic N stage**	495				
N0	327	Reference		Reference	
N1 & N2 & N3	168	2.582 (1.922–3.470)	<0.001	2.238 (1.071–4.677)	0.032
**Pathologic M stage**	363				
M0	338	Reference		Reference	
M1	25	2.143 (1.251–3.672)	0.006	1.822 (0.810–4.097)	0.147
**Pathologic stage**	499				
Stage I	272	Reference		Reference	
Stage II & Stage III & Stage IV	227	2.958 (2.177–4.021)	<0.001	0.858 (0.370–1.991)	0.722
**Primary therapy outcome**	421				
PD & SD & PR	110	Reference		Reference	
CR	311	0.350 (0.249–0.493)	<0.001	0.399 (0.281–0.568)	<0.001
**DUOX1**	507				
High	254	Reference		Reference	
Low	253	1.316 (0.980–1.766)	0.068	1.173 (0.828–1.664)	0.523

### Testing a DUOX1-related nomogram

**Figure 9:**
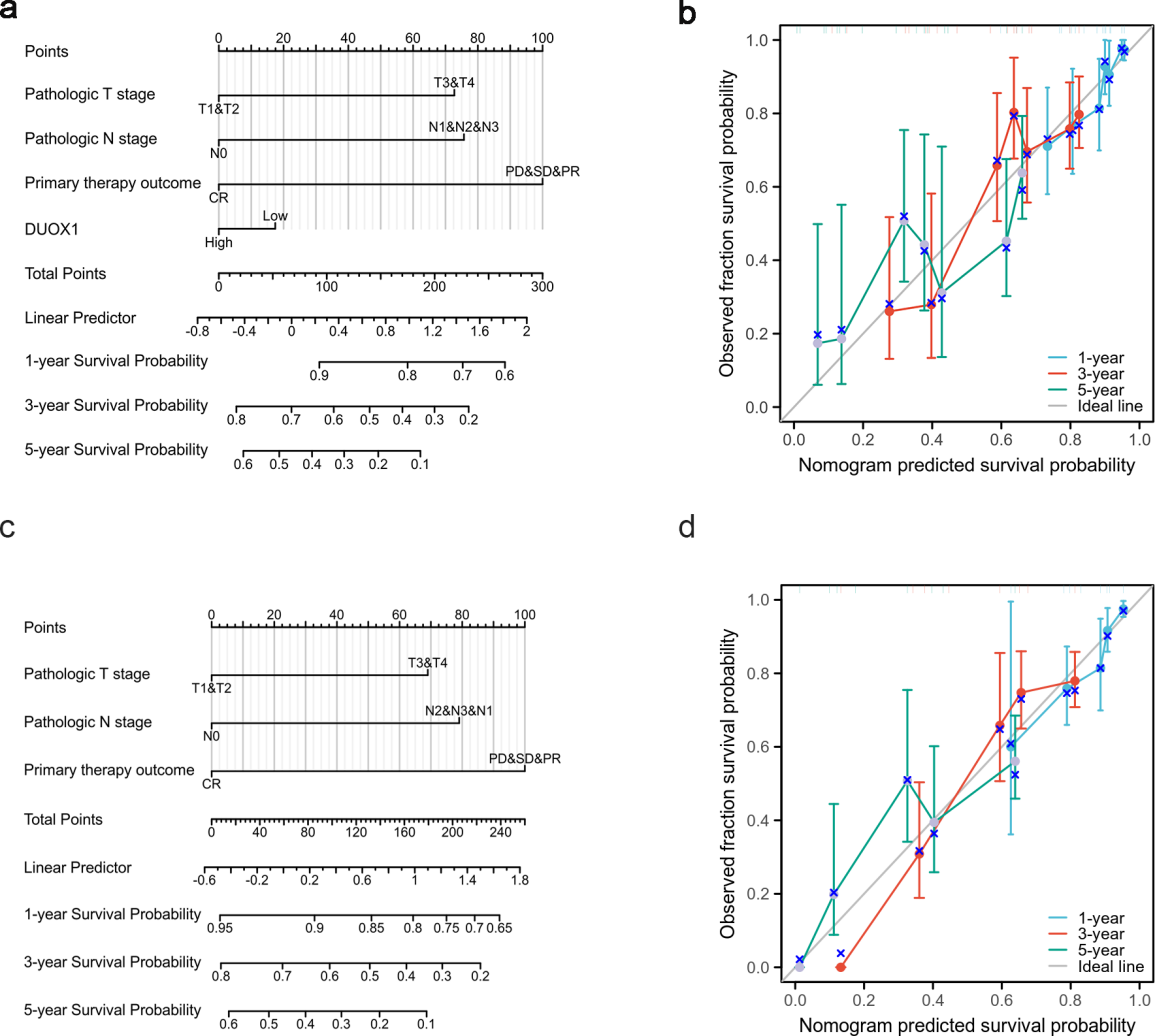
Lung adenocarcinoma prognostic model that predicts 1-, 3-, and 5-year overall survival (OS). The constructed nomograms with DUOX1 (A) and without DUOX1 (C), and nomogram calibration plots with DUOX1 (B) and without DUOX1 (D) were used to calculate the risk of overall survival at 1, 3, and 5 years.

Based on the multivariate analysis, we constructed a prognostic nomogram to predict the prognosis of patients with lung adenocarcinoma based on DUOX1 expression and other independent clinical variables. As shown, the nomogram was used to predict overall survival (OS) at 1, 3, and 5 years in patients with lung adenocarcinoma (Fig. 9A). Additionally, calibration curves were constructed to assess the efficiency of the nomogram (Fig. 9B). The OS lines at 1, 3, and 5 years were close to the ideal line, indicating satisfactory accuracy of the nomogram model. To evaluate the impact of DUOX1 on prognosis, we reconstructed the nomogram excluding DUOX1 while retaining the other three clinicopathological parameters (Figs. 9C, 9D). Using global statistical tests and calibration metrics, we compared the performance of the two nomograms (Table 6). The results showed that the C-index of the nomogram including DUOX1 was 0.684, while the C-index slightly decreased to 0.679 when DUOX1 was excluded.

**Table 6:** Comparison of global statistical test results between the two nomogram models.

**Model characteristics**	**Nomogram with DUOX1**	**Nomogram without DUOX1**
Concordance index (C-index) [95% CI]	0.684 [0.658–0.710]	0.679 [0.654–0.704]
Likelihood ratio test	*χ*^2^= 55.72, *df* = 4, *P* = 3.27 × 10^−13^	*χ*^2^= 54.91, *df* = 3, *P* = 7.17 × 10^−12^
Score (Log-rank) test	*χ*^2^= 64.51, *df* = 4, *P* = 3.27 × 10^−13^	*χ*^2^= 64.04, *df* = 3, *P* = 8.07 × 10^−14^

**Notes.**

df degrees of freedom CI confidence interval

*P*-values <0.05 are considered statistically significant.

## Discussion

The increased detection rate of patients with MPLC is attributable to the development of imaging techniques and early screening for lung cancer. A concern in MPLC management is that sMPLC is difficult to distinguish from IPM. However, the treatment strategies and prognosis for these two tumors are completely different; hence, it this is gradually becoming a research focus. There is currently no gold standard for the diagnosis of MPLC, particularly in patients with similar pathological histological types, which is the subject of some controversy (Tian et al., 2022). With the development of NGS technology, molecular genetic features are expected to assist in the diagnosis of MPLC and facilitate targeted therapy for patients with these types of tumors.

Previous studies have mostly focused on genomics analysis of the clonal origin of each lesion in multifocal lung cancers; that is, each MPLC lesion has a different clonal origin, while tumors with the same clonal origin are classified as IPM (Warth et al., 2012). Before the advent of multi-genomic NGS, researchers demonstrated the unique molecular genetic features of MPLC based on microsatellite instability (Shen et al., 2013), loss of heterozygosity (LOH) analysis (Warth et al., 2012), X-chromosome inactivation analysis (Wang et al., 2009), and array comparative genomic hybridization (Girard et al., 2009) analyses. In the era of high-throughput gene sequencing, NGS-based studies have more comprehensively elucidated the clonal relationships among the lesions of multifocal lung cancers, providing researchers with a deeper understanding of the evolutionary processes leading to metastasis and differences in the primary sources of such tumors (Zhou et al., 2022). This has facilitated the development of targeted therapies for MPLCs. Additionally, due to the high differential rate of driver mutations in MPLCs, patients with MPLCs have different responses to targeted therapy, which is a major challenge for the application of targeted drugs in the treatment of MPLCs. Therefore, further studies are needed to determine the common genetic features of all lesions in an individual, which may facilitate the use of targeted therapy in patients with MPLCs. In this study, transcriptome sequencing analysis was used to analyze MPLCs from the perspective of gene expression to identify common genetic features of MPLC lesions and provide new insights for the diagnosis and treatment of MPLCs.

Some studies have shown that *p53* mutations are characterized by early occurrence, high frequency, uniform distribution, and involvement of a limited number of exons, which may be used as an effective biomarker for the diagnosis of MPLCs (Van Rens et al., 2002; Chang et al., 2019). The *EGFR* and *KRAS* genes are mutation hotspots in lung adenocarcinoma that occur randomly in MPLC. Specifically, *EGFR L858R* mutations occur more frequently in early sMP-LUAD (Pei et al., 2021). The discordance of *EGFR* mutations is higher than that of intrapulmonary metastases (Han et al., 2020), which can be used as a potential biomarker for the differentiation of MPLCs, especially in cases with the same histopathological type (Chang et al., 2019). A combined *EGFR* and *KRAS* mutation pattern analysis improves the diagnostic rate of MPLCs (Takamochi et al., 2012). There is a significant difference in the incidence of combined *EGFR* and *ALK* changes between multifocal lung adenocarcinoma and single-focal lung adenocarcinoma, and an assessment of the relative abundance of *EGFR* mutations and *ALK* rearrangements in patients with multifocal lung adenocarcinoma with co-altered *EGFR/ALK* can distinguish mMPLC from intrapulmonary metastases (Fan et al., 2019). In our study, a total of 194 differentially expressed genes were identified, including 22 up- and 172 down-regulated genes. We found no significant differences in gene expression from hotspot mutated genes between sMP-LUAD and SP-LUAD samples. This may be because the hotspot mutated genes of lung cancer are the broad-spectrum and high-frequency mutated genes of lung adenocarcinoma. The two groups of cancer tissue samples used in this study were both lung adenocarcinoma tissues, and there was no theoretical difference. Additionally, mutation spectrum focuses on mutation frequency and clonal relationships between cancer foci. The expression spectrum is commonly used to explain the molecular regulatory mechanisms of disease and can be used as a biomarker to reflect the disease status of tissue samples; this may also be one of the reasons for the results obtained in this study. This further highlights the importance of analyzing the common genetic characteristics of MPLCs. In the future, we will comprehensively evaluate the genetic characteristics of MPLCs based on DNA sequencing and the mutation spectrum information of MPLCs in public databases, combined with the results of this study.

Several differentially expressed mRNAs in our study were transcribed from cancer-related genes and may be involved in the progression of MPLCs. To elucidate the potential functions of these differentially expressed RNAs, GO functional analysis and KEGG pathway analysis were performed. GO is a bioinformatics concept that unifies genes and gene products across all species; hence, GO functional analysis can be used to annotate and predict the functions of these differentially expressed RNAs in three categories: biological processes, cellular components, and molecular functions. Overall, GO functional analysis showed that the differentially expressed mRNA were significantly related to biological processes including cell adhesion, oxidation–reduction process, and signal transduction. The cellular components were significantly related to membrane, plasma membrane, and integral components of the membrane. Those related to the molecular function had transferase activity and could bind to specific substances (proteins, metal ions, or identical proteins). Therefore, we speculate that these differentially expressed mRNAs may be related to the growth, development, proliferation, metabolism, and transduction of related signals of tumor cells and may promote the occurrence and development of MPLCs from multiple perspectives. KEGG analysis showed that differentially expressed genes were enriched in the PI3K-Akt signaling pathway. The PI3K-Akt pathway is an important intracellular signaling pathway in the cell cycle and is related to cell quiescence, proliferation, cancer, and longevity (Xie et al., 2019). The activation of PI3K phosphorylates and further activates Akt, which leads to a variety of biological functions, including the activation cAMP response element binding protein, inhibition of p27, and cytoplasmic translocation of forkhead box O (FOXO). The PI3K-Akt pathway is known to be enhanced by several biological molecules, including epidermal growth factor (EGF), insulin, and calmodulin. In contrast, this pathway is antagonized by molecules including phosphatase and tensin homolog (PTEN), glycogen synthase kinase 3*β*, and transcription factor HB9. Therefore, we infer that these differentially expressed mRNAs may play a role in multiple tumor-associated cell signaling pathways. Overall, transcriptome sequencing analysis can be applied to the diagnosis of sMP-LUAD and can facilitate the exploration of the internal molecular mechanism of MPLCs.

In our study, we used qRT-PCR to analyze *DUOX1*, *CACNA2D2*, *GPX8*, *COL1A2*, and *COL1A1* expression, and found that the differential expression of these five genes between sMP-LUAD and SP-LUAD showed a similar trend to that observed in mRNA sequencing data; however, only the differences in *DUOX1* expression between sMP-LUAD and SP-LUAD were statistically significant. DUOX1 is a selective oncogene involved in tumorigenesis and cancer progression and its expression is decreased in lung cancer; however, its mechanism of action *in vivo* remains largely unclear (Ling et al., 2014). Consistent with our findings, a previous study reported that DUOX1 can promote reactive oxygen species (ROS) production and observed a correlation between intracellular ROS accumulation and tumor suppression (Sandiford et al., 2014). ROS can be associated with anti-apoptosis, proliferation, metastasis, and angiogenesis; however, ROS can also have pro-cytotoxic and pro-apoptotic effects that inhibit malignant progression and carcinogenesis, depending on the location and type of tumor (Little et al., 2017). In our study, GO enrichment analysis of *DUOX1* showed that *DUOX1* was enriched in the membrane, cell surface, and integral component of the membrane, and this was closely related to its high expression on the apical membrane of respiratory epithelial cells as a membrane protein (Ling et al., 2014). Regarding biological processes, it was enriched in wound healing, cell motility, and other processes, which were closely related to the invasion and migration phenotypes in lung cancer. Epithelial-mesenchymal transition (EMT) is a key feature of aggressive and metastatic cancers, and the loss of DUOX1 is associated with a decrease in the epithelial marker E-cadherin, indicating that DUOX1 is closely associated with EMT (Little et al., 2016). Recent studies have shown that oxidative stress-related genes play an important role in the progression of lung cancer (Qian et al., 2024; Ullah et al., 2024). As a dual oxidase, DUOX1 may promote redox reactions to inhibit EMT by producing ROS. This depends on our subsequent experiments to verify. In our study, KEGG enrichment of DUOX1 revealed that DUOX was associated with thyroid hormone synthesis. Thyroid hormones not only regulate normal growth, development, and metabolism but also stimulate the proliferation of cancer cells and play a role in the occurrence and development of tumors. A previous study (Krashin et al., 2019) showed that thyroid hormone plays an important role in the growth and angiogenesis of tumor cells in lung cancer and indicated that thyroid hormone levels were related to the severity of lung cancer. These results suggest that DUOX1 is closely related to the malignant phenotype of lung cancer, plays an important role in the occurrence and development of lung cancer, and can be used as a target gene for subsequent experiments.

In this study, TCGA was used to further verify the expression pattern and clinical significance of *DUOX1* mRNA in lung adenocarcinoma. The study has found that compared to normal lung tissue, the expression level of *DUOX1* mRNA shows no significant difference in sMP-LUAD, but is significantly reduced in SP-LUAD. Furthermore, the low expression of DUOX1 occurs at all stages of lung adenocarcinoma, suggesting that the silencing of DUOX1 may promote the progression of SP-LUAD. Furthermore, the expression level of DUOX1 was decreased in different histological subtypes of lung adenocarcinoma and was lower in micropapillary and solid lung adenocarcinoma than in other subtypes. Taken together, these findings indicate that DUOX1 silencing is directly proportional to tumor severity.

In this study, the single-sample gene set enrichment analysis (ssGSEA) method was used to evaluate the correlation between *DUOX1* mRNA expression and the abundance of tumor immune infiltrating cells. The results showed that DUOX1 expression was significantly correlated with the number of mast cells, eosinophils, and Th2 cells. Mast cells can improve the prognosis related to lymph node infiltration through the activation of anti-tumor antibodies (Pascal et al., 2024; Leveque et al., 2022; Guo et al., 2022). Eosinophils enhance E-cadherin expression (Pascal et al., 2024), which may inhibit metastasis and improve the clinical outcome of patients with advanced non-small cell lung cancer (Grisaru-Tal et al., 2020). The recruitment of immune cells mediated by DUOX1 inhibits tumor growth and metastasis by phagocytosing cancer cells. Additionally, the co-production of ROS by DUOX1 helps in killing cancer cells and enhancing the host’s defense (Little et al., 2017; Candel et al., 2014). Low DUOX1 expression is often associated with a poorer prognosis, and logistic regression analysis also verified this finding. These findings help explain why *DUOX1* mRNA expression is inversely associated with the survival outcomes of patients with lung adenocarcinoma. However, it should be noted that the association between tumor immune infiltrating cells and *DUOX1* mRNA expression was based only on TCGA database analysis results, and the tumor immune infiltrating cells correlation coefficient was not high. The complex interaction between tumor immune infiltrating cells and the tumor immune microenvironment of lung adenocarcinoma needs to be further verified and explored.

This study aimed to evaluate the correlation between *DUOX1* mRNA expression and the prognosis of patients with lung adenocarcinoma. In the Kaplan–Meier survival evaluation, low DUOX1 expression indicated shorter overall survival; however, there was no significant correlation between DUOX1 expression and OS, DSS, or PFI. Univariate and multivariate Cox regression analysis also suggested that DUOX1 was associated with the prognosis of patients with lung adenocarcinoma, but it could not be used as an independent and reliable predictor. Therefore, we developed a predictive nomogram that combines clinicopathological variables and *DUOX1* mRNA expression to predict survival outcomes in patients with lung cancer. To further evaluate the impact of DUOX1 on prognosis, we reconstructed the nomogram excluding DUOX1. By comparing the performance of the two nomograms (with and without DUOX1), it was found that both models had *p*-values less than 0.001, indicating that both nomograms possess statistically significant predictive capabilities. After excluding DUOX1, there was a slight decrease in the C-index and a reduction in calibration performance, suggesting that DUOX1 contributes to the prognostic discriminative ability of the model.

This study has some limitations. First, the sample size was small, and confounding factors such as age and smoking history of patients were not considered. Future studies should include a larger sample size and stratified analysis will be carried out. Second, RNA-seq only detects the transcriptome level and does not involve the proteome or metabolome. Future plans include integrating multi-omics technologies to improve the mechanism. Third, this study is a single-center study, which may have regional limitations. We have partially validated this issue through the TCGA database. In addition, although we analyzed the biological function and clinical significance of DUOX1 in lung adenocarcinoma, it has not been experimentally verified. We will clarify its specific mechanism through subsequent cell experiments. In the future, transcriptome biomarkers can be combined with liquid biopsies to develop test kits for the precise diagnosis of sMP-LUAD.

In conclusion, using a series of sMP-LUAD and SP-LUAD cases, we found there were differentially expressed genes in the transcriptome profiles in lung cancer between the two groups, based on transcriptome analysis. This suggests that transcriptome sequencing may be a new approach for the diagnosis of multiple primary lung adenocarcinoma. Additionally, differentially expressed genes were verified at the tissue level using qRT-PCR, and the differential gene *DUOX1* mRNA was screened out. Furthermore, GO enrichment analysis identified that DUOX1 may be associated with EMT, which is a key feature of aggressive and metastatic cancers, suggesting that DUOX1 may be associated with the malignant phenotype of multiple primary lung cancers. Furthermore, based on the TCGA database, we analyzed the biological behavior and clinical significance of DUOX1 in lung adenocarcinoma using bioinformatics technology. We found that DUOX1 was lowly expressed in lung adenocarcinomas, and its expression level was lower in micropapillary and solid lung adenocarcinomas than in other subtypes. This may indicate that DUOX1 silencing is directly proportional to tumor severity. DUOX1 also affected OS; however, its effect was not significant and could not be used as an independent prognostic factor. Furthermore, we developed a predictive nomogram model combining clinicopathological variables and *DUOX1* mRNA to predict the survival of patients with lung adenocarcinoma.

## Conclusions

Differentially expressed genes exist in the transcriptome profiles between sMP-LUAD and SP-LUAD, suggesting that transcriptome sequencing may provide a new method for the diagnosis of multiple primary lung adenocarcinomas. Functional enrichment analysis of *DUOX1* mRNA suggests that it may be related to epithelial-mesenchymal transition and participates in the malignant biological phenotype of multiple primary lung cancers; however, it is not an independent predictor of prognosis.

## Supplemental Information

10.7717/peerj.20617/supp-1 Supplemental Information 1 Raw data for qRT-PCR

10.7717/peerj.20617/supp-2 Supplemental Information 2 The raw data of transcriptome sequencing analysis about MPLC and SP-LUAD

10.7717/peerj.20617/supp-3 Supplemental Information 3 The raw data of RNA-seq

10.7717/peerj.20617/supp-4 Supplemental Information 4 General clinical data with sMP-LUAD and SP-LUAD The raw data for Table 2.

10.7717/peerj.20617/supp-5 Supplemental Information 5 STROBE checklist

10.7717/peerj.20617/supp-6 Supplemental Information 6 MIQE checklist

## Additional Information and Declarations

### Data Availability

The following information was supplied regarding data availability: The raw measurements are available in the Supplementary Files.
