# Supplementary material for: Molecular characterization of early-stage multi-primary lung adenocarcinoma by transcriptome sequencing—a retrospective study
Source: PeerJ. 2026 Jan 15;14:e20617. doi: 10.7717/peerj.20617 (PMC12812280; doi:10.7717/peerj.20617)
Supplement: Supplemental Information 5 [file peerj-14-20617-s005.docx]

STROBE Statement—checklist of items that should be included in reports of observational studies

|  | Item No. | Recommendation | Page  No. | Relevant text from manuscript |
| --- | --- | --- | --- | --- |
| **Title and abstract** | 1 | (*a*) Indicate the study’s design with a commonly used term in the title or the abstract | 1 | A retrospective study |
|  |  | (*b*) Provide in the abstract an informative and balanced summary of what was done and what was found | 1 | Lines 22-33 |
| Introduction | | | |  |
| Background/rationale | 2 | Explain the scientific background and rationale for the investigation being reported | 2 | Lines 61-73 |
| Objectives | 3 | State specific objectives, including any prespecified hypotheses | 3 | Lines 73-79 |
| Methods | | | |  |
| Study design | 4 | Present key elements of study design early in the paper | 3 | Lines 74-76 |
| Setting | 5 | Describe the setting, locations, and relevant dates, including periods of recruitment, exposure, follow-up, and data collection | 3 | Lines 86-88 |
| Participants | 6 | (*a*) *Cohort study*—Give the eligibility criteria, and the sources and methods of selection of participants. Describe methods of follow-up  *Case-control study*—Give the eligibility criteria, and the sources and methods of case ascertainment and control selection. Give the rationale for the choice of cases and controls  *Cross-sectional study*—Give the eligibility criteria, and the sources and methods of selection of participants | 3 | Lines 89-94 |
|  |  | (*b*) *Cohort study*—For matched studies, give matching criteria and number of exposed and unexposed  *Case-control study*—For matched studies, give matching criteria and the number of controls per case | - | - |
| Variables | 7 | Clearly define all outcomes, exposures, predictors, potential confounders, and effect modifiers. Give diagnostic criteria, if applicable | 2 | Lines 48-60 |
| Data sources/ measurement | 8* | For each variable of interest, give sources of data and details of methods of assessment (measurement). Describe comparability of assessment methods if there is more than one group | 3 | Lines 83-99 |
| Bias | 9 | Describe any efforts to address potential sources of bias | 3 | Lines 83-99.  Sample selection is homogenized. |
| Study size | 10 | Explain how the study size was arrived at | 2 | Lines 74-76.  This study was an RNA sequencing study, so the minimum sample size needed for measurement data was selected. |

Continued on next page

| Quantitative variables | 11 | Explain how quantitative variables were handled in the analyses. If applicable, describe which groupings were chosen and why | 3,4 | Lines 109-127 |
| --- | --- | --- | --- | --- |
| Statistical methods | 12 | (*a*) Describe all statistical methods, including those used to control for confounding | 4 | Lines 140-146 |
|  |  | (*b*) Describe any methods used to examine subgroups and interactions | 4 | Lines 143-145 |
|  |  | (*c*) Explain how missing data were addressed | 4 | Lines 145-146 |
|  |  | (*d*) *Cohort study*—If applicable, explain how loss to follow-up was addressed  *Case-control study*—If applicable, explain how matching of cases and controls was addressed  *Cross-sectional study*—If applicable, describe analytical methods taking account of sampling strategy |  | Not involve |
|  |  | (*e*) Describe any sensitivity analyses | 4 | Lines 145-146 |
| Results | | | | |
| Participants | 13* | (a) Report numbers of individuals at each stage of study—eg numbers potentially eligible, examined for eligibility, confirmed eligible, included in the study, completing follow-up, and analysed | 4 | Lines 150-153  Eight patients with sMP-LUAD and eight patients with SP-LUAD |
|  |  | (b) Give reasons for non-participation at each stage |  | Not involve |
|  |  | (c) Consider use of a flow diagram |  | Table 2 |
| Descriptive data | 14* | (a) Give characteristics of study participants (eg demographic, clinical, social) and information on exposures and potential confounders | 4,5 | Lines 150-160 |
|  |  | (b) Indicate number of participants with missing data for each variable of interest |  | Not involve |
|  |  | (c) *Cohort study*—Summarise follow-up time (eg, average and total amount) |  | - |
| Outcome data | 15* | *Cohort study*—Report numbers of outcome events or summary measures over time |  | *-* |
|  |  | *Case-control study—*Report numbers in each exposure category, or summary measures of exposure | *5* | Lines 163-167 |
|  |  | *Cross-sectional study—*Report numbers of outcome events or summary measures |  | *-* |
| Main results | 16 | (*a*) Give unadjusted estimates and, if applicable, confounder-adjusted estimates and their precision (eg, 95% confidence interval). Make clear which confounders were adjusted for and why they were included | 5 | Lines 161-182 |
|  |  | (*b*) Report category boundaries when continuous variables were categorized |  | - |
|  |  | (*c*) If relevant, consider translating estimates of relative risk into absolute risk for a meaningful time period |  | - |

Continued on next page

| Other analyses | 17 | Report other analyses done—eg analyses of subgroups and interactions, and sensitivity analyses | 5 | Lines 183-192 |
| --- | --- | --- | --- | --- |
| Discussion | | | | |
| Key results | 18 | Summarise key results with reference to study objectives | 9 | Lines 323-329 |
| Limitations | 19 | Discuss limitations of the study, taking into account sources of potential bias or imprecision. Discuss both direction and magnitude of any potential bias | 8 | Lines 306-312 |
| Interpretation | 20 | Give a cautious overall interpretation of results considering objectives, limitations, multiplicity of analyses, results from similar studies, and other relevant evidence | 9 | Lines 313-320 |
| Generalisability | 21 | Discuss the generalisability (external validity) of the study results | 9 | Lines 323-329 |
| Other information | |  | | |
| Funding | 22 | Give the source of funding and the role of the funders for the present study and, if applicable, for the original study on which the present article is based |  | Academician (expert) workstation project (202305AF140274) |

*Give information separately for cases and controls in case-control studies and, if applicable, for exposed and unexposed groups in cohort and cross-sectional studies.

**Note:** An Explanation and Elaboration article discusses each checklist item and gives methodological background and published examples of transparent reporting. The STROBE checklist is best used in conjunction with this article (freely available on the Web sites of PLoS Medicine at http://www.plosmedicine.org/, Annals of Internal Medicine at http://www.annals.org/, and Epidemiology at http://www.epidem.com/). Information on the STROBE Initiative is available at www.strobe-statement.org.
